# Supplementary material for: Modelling the impact of fexinidazole use on human African trypanosomiasis (HAT) transmission in the Democratic Republic of the Congo
Source: PLoS Negl Trop Dis. 2021 Nov 29;15(11):e0009992. doi: 10.1371/journal.pntd.0009992 (PMC8659363; doi:10.1371/journal.pntd.0009992)
Supplement: S1 Text — Description of model equations, parameter values, and fitting methods. (PDF) [file pntd.0009992.s001.pdf]

Supplementary information

Modelling the impact of fexinidazole use on human African trypanosomiasis (HAT) transmission in the Democratic Republic of Congo

Aatreyee M. Das[1,2], Nakul Chitnis[1,2], Christian Burri[1,2], Daniel H. Paris[1,2], Swati Patel[3,4], Simon E.F. Spencer[3], Erick M. Miaka[5], M. Soledad Castaño[1,2]

1 Department of Epidemiology and Public Health,
Swiss Tropical and Public Health Institute, Basel, Switzerland
2 University of Basel, Basel, Switzerland
3 Department of Statistics, University of Warwick, Coventry, CV4 7AL, United Kingdom
4 Department of Mathematics, Oregon State University, Corvallis, United States
5 Programme National de Lutte contre la Trypanosomiase Humaine Africaine,
Kinshasa, the Democratic Republic of the Congo

1 Model description

1.1 Deterministic model

This description is adapted from [1, Supplementary data].

The deterministic model used here was presented and described in [2] and is a variant of the HAT transmission model originally published in [3] and [1]. The model consists of a system of coupled ordinary differential equations (ODEs), with compartments for tsetse, non-human and human populations. These three different host types are modelled for two different settings corresponding to a low transmission area (e.g. the village, L) and a high transmission area (such as river banks or plantations, H) that enable accounting for heterogeneity in exposure to tsetse bites. The population size for tsetse, non-human hosts or humans in each setting i ($i = \{L, H\}$) is assumed to be stable by allowing the associated birth terms to compensate deaths in all the compartments. Tsetse and non-human host populations always stay within their setting (for example, tsetse in low transmission settings always remain in the low transmission setting and non-human hosts in high transmission settings always remain in the high transmission setting). Similarly, humans in low transmission settings always remain in low transmission setting. However, humans in the high transmission setting move back and forth between the high and low transmission settings spending a fixed amount of time in each one (to model, for example, the movement of high risk individuals between villages and plantations) — as shown in Figure 1.

Five compartments describe humans in any of the two settings: susceptible (S_{hi}); exposed or incubating (E_{hi}); infected with the first stage of the disease (I_{h1i}); infected with the second stage of the disease, where trypanosomes have reached the cerebro-spinal fluid

(I_{h2i}); and treated (T_{hi}). The total human population in setting i is $N_{hi} = S_{hi} + E_{hi} + I_{h1i} + I_{h2i} + T_{hi}$. Humans can simultaneously belong in the diagnosed compartment (D_{hi}) and one of the infected stages, from which, depending on drug compliance, they may either move on to the treated compartment, or remain in the infected compartment.

Tsetse populations are divided into susceptible (S_{vi}); teneral (U_{vi}); exposed (E_{vi}); and infected (I_{vi}), so that the vector population is $N_{vi} = S_{vi} + U_{vi} + E_{vi} + I_{vi}$.

As in [1], in this model implementation: *i)* non-human hosts do not contribute to transmission, thus non-human host populations are modelled as constant parameters, N_{ai}, and only form a sink for tsetse bite; *ii)* both stages (rather than only stage 1) of the disease are exposed to tsetse fly bites; *iii)* an additional compartment in the vector dynamics, U_i, accounts for the teneral effect — a reduction of infectivity with time — such that on average tsetse are only infectious for the first five days after emergence. These changes were made with respect to the original version [3] to provide a more realistic representation of the transmission dynamics. A schematic of the model is shown in Figure 1.

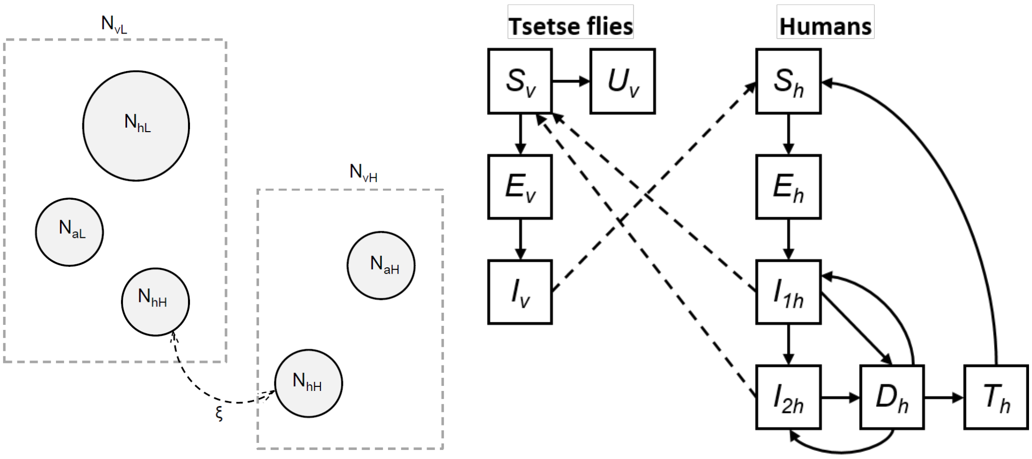

Figure 1: Schematic of the model. Left: model population structure. Human populations are composed by a stationary population (N_{hL}) that remains in low exposure habitats (e.g., a village), and a smaller population (N_{hH}) which commute and spend a proportion ξ of their time in a potentially high exposure setting (e.g., a plantation). Each habitat also contains tsetse (N_{vL} and N_{vH}) and non-human vertebrate animal populations (N_{aL} and N_{aH}). Right: schematic of infection dynamics, subscripts $i = \{L, H\}$ were removed for easy reading. Compartmental diagram highlights the transmissions between states of infection of the tsetse and human populations, with solid lines indicating transition between compartments, and dashed lines representing transmission rates. Non-human hosts cannot transmit infection thus acting as a sink for tsetse bite. Note that in the low-risk transmission setting, both human populations are exposed to tsetse bites. Figure adapted from [3].

1.1.1 Model equations

The model dynamics are described by sets of ODEs for humans, vectors and non-human hosts. Descriptions of state variables and parameters can be found in Tables 1, 2 and 4. A description of r_{as} and r_{pd} can be found in section1.2.

Humans, low risk setting:

$$
\begin{aligned}
\frac{dS_L}{dt} &= \beta_L + \delta T_L - \mu S_L - \lambda_L I_{v_L} S_L \\
\frac{dE_L}{dt} &= \lambda_L I_{v_L} S_L - (\mu + \eta) E_L \\
\frac{dI_{1L}}{dt} &= \eta E_L - (\mu + \gamma) I_{1L} - (r_{as} + r_{1pd}) I_{1L} + (1 - \psi)(r_{as} + r_{1pd}) \phi_1 I_{1L} \\
\frac{dI_{2L}}{dt} &= \gamma I_{1L} - (\mu + \mu_\gamma) I_{2L} - (r_{as} + r_{2pd}) I_{2L} + (1 - e\psi)(r_{as} + r_{2pd}) \phi_2 I_{2L} \\
\frac{dD_{1L}}{dt} &= (r_{as} + r_{1pd}) I_{1L} - \psi (r_{as} + r_{1pd}) \phi_1 I_{1L} - (r_{as} + r_{1pd})(1 - \phi_1) I_{1L} \\
&\quad - (1 - \psi)(r_{as} + r_{1pd}) \phi_1 I_{1L} \\
\frac{dD_{2L}}{dt} &= (r_{as} + r_{2pd}) I_{2L} - e\psi (r_{as} + r_{2pd}) \phi_2 I_{2L} - (r_{as} + r_{2pd})(1 - \phi_2) I_{2L} \\
&\quad - (1 - e\psi)(r_{as} + r_{2pd}) \phi_2 I_{2L} \\
\frac{dT_L}{dt} &= \psi (r_{as} + r_{1pd}) \phi_1 I_{1L} + (r_{as} + r_{1pd})(1 - \phi_1) I_{1L} + e\psi (r_{as} + r_{2pd}) \phi_2 I_{2L} \\
&\quad + (r_{as} + r_{2pd})(1 - \phi_2) I_{2L} - (\mu + \mu_t + \delta) T_L
\end{aligned}
$$

where

- ϕ_i (for either low-risk or high-risk setting) is the proportion (range to determine between 0 and 1) of cases treated with fexinidazole (i.e. those who do not fall into the following categories: children < 6 years; patients weighing < 20kg; pregnant women in the first trimester; advanced stage 2 cases). $\phi_1 > \phi_2$ as advanced stage 2 patients must follow the current treatment.
- ψ (for either low-risk or high-risk setting) is the proportion of cases the comply with fexinidazole treatment sufficiently to count as full compliance.
- e is the relatively reduction in fexinidazole efficacy for treatment of stage 2 HAT as compared to NECT.
- For both active screening and passive detection, we assume perfect compliance to the current first-line treatment, the lesser compliance to fexinidazole is represented by a factor ψ between 0 and 1 such that this proportion of patients receiving fexinidazole comply with treatment and moved to the treated stage (same for stage 2). The remaining $1 - \psi$ proportion of patients treated with fexinidazole do not comply and remain infected despite having been diagnosed.

Related to the force of infection,

$$\lambda_L = \frac{bf\theta_{v_L h_L}}{N_L}$$

where the probability of biting a human for the vector population in the low risk setting, N_{vL}, can be split in two: $\theta_{v_L h_L}$ and $\theta_{v_L h_H}$, defined as:

$$\theta_{v_L h_L} = \frac{\sigma N_L}{\sigma(N_L + (1 - \xi) N_H) + \sigma_{aL} N_{aL}}$$

$$\theta_{v_L h_H} = \frac{\sigma(1-\xi)N_H}{\sigma(N_L + (1-\xi)N_H) + \sigma_{aL}N_{aL}},$$

and the probability of biting a non-human host for the vector population in the low risk setting is:

$$\theta_{v_L a_L} = \frac{\sigma_{aL}N_{aL}}{\sigma(N_L + (1-\xi)N_H) + \sigma_{aL}N_{aL}}.$$

Note that

$$\beta_L = \mu(S_L + E_L + I_{1L} + I_{2L} + T_L) + \mu_\gamma I_{2L} + \mu_t T_L.$$

and that N_L indicates the part of the population exposed to bites,

$$N_L = S_L + E_L + I_{1L} + I_{2L}.$$

Humans, high risk setting:

$$\begin{aligned}
\frac{dS_H}{dt} &= \beta_H + \delta T_H - \mu S_H - \lambda_{H1} I_{v_L} S_H - \lambda_{H2} I_{v_H} S_H \\
\frac{dE_H}{dt} &= \lambda_{H1} I_{v_L} S_H + \lambda_{H2} I_{v_H} S_H - (\mu + \eta) E_H \\
\frac{dI_{1H}}{dt} &= \eta E_H - (\mu + \gamma) I_{1H} - r_{1pd} I_{1H} + (1-\psi) r_{1pd}\phi_1 I_{1H} \\
\frac{dI_{2H}}{dt} &= \gamma I_{1H} - (\mu + \mu_\gamma) I_{2H} - r_{2pd} I_{2H} + (1-e\psi) r_{2pd}\phi_2 I_{2H} \\
\frac{dD_{1H}}{dt} &= r_{1pd} I_{1H} - \psi r_{1pd}\phi_1 I_{1H} - r_{1pd}(1-\phi_1) I_{1H} - (1-\psi) r_{1pd}\phi_1 I_{1H} \\
\frac{dD_{2H}}{dt} &= r_{2pd} I_{2H} - e\psi r_{2pd}\phi_2 I_{2H} - r_{2pd}(1-\phi_2) I_{2H} - (1-e\psi) r_{2pd}\phi_2 I_{2H} \\
\frac{dT_H}{dt} &= \psi r_{1pd}\phi_1 I_{1H} + r_{1pd}(1-\phi_1) I_{1H} + e\psi r_{2pd}\phi_2 I_{2H} + r_{2pd}(1-\phi_2) I_{2H} \\
&\quad - (\mu + \mu_t + \delta) T_H
\end{aligned}$$

with

$$\begin{aligned}
\lambda_{H1} &= \frac{bf\theta_{v_L h_H}}{N_H} \\
\lambda_{H2} &= \frac{bf\theta_{v_H h_H}}{N_H}.
\end{aligned}$$

where the probability of biting a human for the vector population in the high risk setting, N_{vH}, is $\theta_{v_H h_H}$:

$$\theta_{v_H h_H} = \frac{\sigma\xi N_H}{\sigma\xi N_H + \sigma_{aH}N_{aH}},$$

and the probability of biting a non-human host for the vector population in the high risk setting is $\theta_{v_H a_H}$:

$$\theta_{v_H a_H} = \frac{\sigma_{aH} N_{aH}}{\sigma \xi N_H + \sigma_{aH} N_{aH}}.$$

Note that

$$\beta_H = \mu N_H + \mu_\gamma I_{2H} + \mu_t T_H.$$

Non-human hosts, low risk setting:

$$\frac{dS_{aL}}{dt} = \beta_{aL} + \delta_a R_{aL} - \mu_{aL} S_{aL} - \lambda_{aL} I_{v_L} S_{aL} \tag{1}$$
$$\frac{dE_{aL}}{dt} = \lambda_{aL} I_{v_L} S_{aL} - (\mu_{aL} + \eta) E_{aL} \tag{2}$$
$$\frac{dI_{aL}}{dt} = \eta E_{aL} - (\mu_{aL} + \gamma_{aL}) I_{aL} \tag{3}$$
$$\frac{dR_{aL}}{dt} = \gamma_{aL} I_{aL} - (\mu_{aL} + \delta_a) R_{aL} \tag{4}$$

with

$$\lambda_{aL} = \frac{b f \theta_{v_L a_L} c_{aL}}{N_{aL}}.$$

Non-human hosts, high risk setting:

$$\frac{dS_{aH}}{dt} = \beta_{aH} + \delta_a R_{aH} - \mu_{aH} S_{aH} - \lambda_{aH} I_{v_H} S_{aH} \tag{5}$$
$$\frac{dE_{aH}}{dt} = \lambda_{aH} I_{v_H} S_{aH} - (\mu_{aH} + \eta) E_{aH} \tag{6}$$
$$\frac{dI_{aH}}{dt} = \eta E_{aH} - (\mu_{aH} + \gamma_{aH}) I_{aH} \tag{7}$$
$$\frac{dR_{aH}}{dt} = \gamma_{aH} I_{aH} - (\mu_{aH} + \delta_a) R_{aH} \tag{8}$$

with

$$\lambda_{aH} = \frac{b f \theta_{v_H a_H} c_{aH}}{N_{aH}}.$$

Note, $c_{aL} = c_{aH} = 0$ so non-human hosts can receive bites, but do not become infected.

Vectors, low risk setting:

$$\frac{dS_{vL}}{dt} = \beta_{vL} - \mu_v S_{vL} - w_{LL}(I_{1L} + I_{2L})S_{vL} - w_{LH}(I_{1H} + I_{2H})S_{vL} - w_{aL}I_{aL}S_{vL} - \alpha S_{vL} \tag{9}$$

$$\frac{dE_{vL}}{dt} = w_{LL}(I_{1L} + I_{2L})S_{vL} + w_{LH}(I_{1H} + I_{2H})S_{vL} + w_{aL}I_{aL}S_{vL} - (\mu_v + \nu)E_{vL} \tag{10}$$

$$\frac{dI_{vL}}{dt} = \nu E_{vL} - \mu_v I_{vL} \tag{11}$$

$$\frac{dU_{vL}}{dt} = \alpha S_{vL} - \mu_v U_{vL} \tag{12}$$

with

$$w_{LL} = f\theta_{v_L h_L}\frac{1}{N_L}c_h \tag{13}$$

$$w_{LH} = f\theta_{v_L h_H}\frac{1}{N_H}c_h \tag{14}$$

$$w_{aL} = c_{aL}f\theta_{v_L a_L}\frac{1}{N_{aL}}. \tag{15}$$

Note: currently $c_h = c_1 = c_2$ so tse-tse have no particular preference on biting stage I or II infected humans.

Vectors, high risk setting:

$$\frac{dS_{vH}}{dt} = \beta_{vH} - \mu_{vH} S_{vH} - w_{HH}(I_{1H} + I_{2H})S_{vH} - w_{aH}I_{aH}S_{vH} - \alpha S_{vH} \tag{16}$$

$$\frac{dE_{vH}}{dt} = w_{HH}(I_{1H} + I_{2H})S_{vH} + w_{aH}I_{aH}S_{vH} - (\mu_v + \nu)E_{vH} \tag{17}$$

$$\frac{dI_{vH}}{dt} = \nu E_{vH} - \mu_v I_{vH} \tag{18}$$

$$\frac{dU_{vH}}{dt} = \alpha S_{vH} - \mu_v U_{vH} \tag{19}$$

with

$$w_{HH} = f\theta_{v_H h_H}\frac{1}{N_H}c_h \tag{20}$$

$$w_{aH} = c_{aH}f\theta_{v_H a_H}\frac{1}{N_{aH}}. \tag{21}$$

1.1.2 Stochastic implementation

This description is adapted from [1, Supplementary data].

Epidemiological deterministic models, including previous implementations of this model [3, 4, 1], have the shortcoming that they do not capture rare events and do no account for

the discrete nature of populations, and are therefore unable to reproduce the transition between extremely low prevalence and zero transmission. For such situations, a discrete stochastic model formulation is more suitable as it captures the stochastic nature of events involved in transmission dynamics while producing integer outputs (e.g. number of cases and new infections here) that enable a clearer definition of elimination of transmission (and subsequent forecasting of elimination timelines) than in deterministic ODE models where arbitrary thresholds must be defined.

In the stochastic formulation of the ODE model described in 1.1, we model all human host, non-human host and tsetse fly populations as discrete numbers, and all individuals move probabilistically between compartments at varying intervals of time. Any process governing the HAT transmission dynamics is considered stochastic, with terms in the compartmental model being now considered as probabilities at which an event occurs.

We implemented the direct method of the stochastic simulation algorithm (SSA; also known as Gillespie method [5]). In the direct method of the SSA, all possible events in the HAT transmission dynamics have an associated rate given by the associated term in the deterministic ODEs. For example, if γ represents the rate at which humans infected in the stage 1 of the disease (I_1) move to the second stage of the disease (I_2), thus γI_1 represents the rate R for the event "progression to stage 2 of the disease".

In order to simulate one stochastic realisation under the direct method, for i possible events with associated rate R_i, at any time t:
(a) we determine the time $t + \tau$ at which the next event happens, with τ an exponentially distributed random number scaled by the sum of all process rates, $\Sigma_i R_i$; and
(b) we decide which that event will be: the event that happens next is obtained through drawing a process randomly from all possible processes according to their respective probabilities given by $R_i / \sum_j R_j$.

In the present analysis, 1000 posterior parameter sets (see section 2) were used along with the fixed parameters to obtain 100,000 realisations of the stochastic model (100 realisations for each parameter set).

Simulations up to 2000 were deterministic, i.e. by numerical solving the ODE system, with further projections using the stochastic implementation described in this section.

The deterministic model output at 2000 was scaled up to follow the population growth trajectory of Mushie territory and rounded to integer values before running stochastic simulations of the forward discrete model. Simulations from 2000 to 2018 were run using parameters calibrated to data, and then projected forward until 2040 including reductions in the active screening rate (see section 1.2) and maintaining the passive detection rate seen in 2018.

The discrete stochastic model was run until 2040 in order to allow evaluation of elimination of transmission (EOT) (Figure 4 in the main text), with EOT defined as the point where there are no exposed or infected humans or vectors present in the simulation.

1.2 Screening

This description is adapted from [2, Supplementary data].

Active screening was modelled via a constant annual active detection rate r_{as} that removes infected people only from the low risk setting. As in previous works, we followed [6] to relate a proportion, d, of humans effectively screened in a given year and the annual

removal rate r_{as} as $d = 1 - e^{-r_{as}}$, leading to $r_{as} = -\ln(1-d)$. For the model fitting, screening levels were informed from data, and estimates for the health zone population in 2015 were taken from [7], and projected backwards and forward in time assuming a 3% annual growth rate. For model projections, the mean number of people screened from the last 5 years of available data (2014-2018) was used to define d, with an ongoing 3% growth rate in the total population (leading to a continuing decrease in the proportion of the population screened).

Passive detection is represented by a continuous stage-specific detection rate, $r1$ and $r2$ for stage 1 and stage 2 respectively, and removes infected people from both low- and high-risk settings. Relying on previous work [1], improvement to passive detection was assumed for the data period. We modelled improvement for the number of years $y > 0$ after 2000 as a logistic function:

$$r1(y) = 365 \times r1_{\text{const}} + \frac{\Delta r_1}{1 + \exp(-\alpha_{\text{pd}}(y - x_0 + 1))},$$

$$r2(y) = 365 \times r1_{\text{const}} \times c2 + \frac{\Delta r_1 + \Delta r_2}{1 + \exp(-\alpha_{\text{pd}}(y - x_0 + 1))},$$

where $r1_{\text{const}}$ and $c_2 \times r1_{\text{const}}$ are the constant daily passive detection rates in stage 1 and stage 2 respectively for any time before 2000, and Δr_1, Δr_2, α_{pd} and x_0 are parameters defining the profile of the logistic curve. All parameters in the expression above are fitted to the health zone level data. For model projections, we assumed passive detection rates $r1$ and $r2$ continue at the highest level from 2000-2018.

Additionally, due to changes to the method of confirming positive HAT cases, including video evidence of moving parasites, a perfect specificity of 100% was assumed from 2017 onwards. This is reflected in the cases seen, as there is a drop in reported cases observed from 2017 onwards.

Our model assumes that before 2000 only passive detection was ongoing, at constant rates, and that active screening activities started in 2000, the initial year for which there is available data on active screening.

2 Fitting procedure

Twelve parameters were fitted using annual case data from Mushie territory for the period 2000–2018. The deterministic model was first run to reach equilibrium prevalence of infection assuming only constant passive screening before 2000, when the fitting starts. The data that was fitted separated reported cases into those from active screening and passive detection, and provided information on staging in the years from 2015 onward. Fitting was performed via an adaptive Metropolis-Hastings Markov chain Monte Carlo (MCMC) approach using the following log-likelihood function:

$$\begin{aligned}
LL(\theta|x) = &\ \log(P(x|\theta)) \\
\propto &\sum_{i=2000}^{2018} \Bigg(\log\left[\text{NegBin}\left(A_{d1}(i) + A_{d2}(i); A_{m1}(i) + A_{m2}(i), \kappa_{\text{AS}}\right)\right] \\
&+ \log\left[\text{NegBin}\left(P_{d1}(i) + P_{d2}(i); P_{m1}(i) + P_{m2}(i), \kappa_{\text{PD}}\right)\right] \Bigg) \\
&+ \sum_{i=2015}^{2018} \Bigg(\log\left[\text{Bin}\left(P_{d1}(i); P_{d1}(i) + P_{d2}(i), \frac{P_{m1}(i)}{P_{m1}(i) + P_{m2}(i)}\right)\right] \\
&+ \log\left[\text{Bin}\left(A_{d1}(i); A_{d1}(i) + A_{d2}(i), \frac{A_{m1}(i)}{A_{m1}(i) + A_{m2}(i)}\right)\right] \Bigg),
\end{aligned}$$

where A_{d1}: stage 1 reported cases (active screening); A_{d2}: stage 2 reported cases (active screening); P_{d1}: stage 1 reported cases (passive detection); P_{d2}: stage 2 reported cases (passive detection); A_{m1}: stage 1 reported cases from the model (active screening); A_{m2}: stage 2 reported cases from the model (active screening); P_{m1}: stage 1 reported cases from the model (passive surveillance); P_{m2}: stage 2 reported cases from the model (passive surveillance); κ_{AS}: shape parameter for the negative binomial distribution for annual number of cases detected through active screening; and κ_{PD}: shape parameter for the negative binomial distribution for annual number of cases detected through passive detection.

The two terms in the first sum represent the total number of active and passive cases in the data, respectively, modeled as a negative binomial with mean equal to the number of cases from the differential equation model. The two terms in the second sum represent the proportion of cases in stage 1, modeled as a binomial in which the probability of stage 1 is the proportion from the differential equation model (and the number of trials is from the data of total number of cases). Since we only have staged data from 2015 onwards, those terms only contribute to the log likelihood in those years.

To sample from the posterior distribution, determined by the likelihood function and the prior distributions, we used an adaptive Metropolis-Hastings MCMC algorithm – the accelerated shaping algorithm [8]. We ran two independent chains of the algorithm to corroborate convergence for the sampling. We used a burn-in period of 2000 steps and then ran the chain for 20,000 steps, which was thinned to every other sample. For the proposal distribution, we used a multivariate Normal distribution (truncated with the bounds given in Table 3), with a covariance matrix that adapts to predict the shape and scale of the posterior distribution as the algorithm proceeds. The adaptation improves the efficiency of proposing new samples so that there are neither excessive rejections nor acceptances in the algorithm. Finally, to improve mixing further, we used the two covariance matrices from this first set of runs in a second round of two independent chains with the same burn-in and sampling strategy. With this second set, we visually checked that there was good mixing. The parameters used in the forward projections are based on the first chain of this second set of runs.

2.1 Fixed parameters, priors and posterior distributions

Descriptions of state variables are given in Table 1. Descriptions and values of all fixed parameters are given in Table 2, and descriptions and prior distributions for all fitted parameters are given in Table 3 and Table 4 respectively.

Notation	Description
S_i	Number of susceptible humans in risk setting i
E_i	Number of exposed humans in risk setting i
I_{1i}	Number of infected humans in stage 1 in risk setting i
I_{2i}	Number of infected humans in stage 2 in risk setting i
D_{1i}	Number of diagnosed humans in stage 1 in risk setting i
D_{2i}	Number of diagnosed humans in stage 1 in risk setting i
T_i	Number of treated humans in risk setting i
S_{ai}	Number of susceptible non-human hosts in risk setting i
E_{ai}	Number of exposed non-human hosts in risk setting i
I_{ai}	Number of infected non-human hosts in risk setting i
R_{ai}	Number of removed non-human hosts in risk setting i
S_{vi}	Number of teneral vectors in risk setting i
E_{vi}	Number of exposed vectors in risk setting i
I_{vi}	Number of infected vectors in risk setting i
U_{vi}	Number of non-teneral vectors in risk setting i

Table 1: State variables in the model. Notation and a brief description of the state variables. $i = L, H$, representing the low and high risk settings respectively.

Parameter	Unit	Prior Distribution and Bounds
κ	-	$\text{Unif}[0, 1]$
log(VHL)	-	N$(1.1, 0.05)$ in [0,log(100)]
log(c_1)	-	Gamma$(1, 1)$ in [0,log(50)]
logit(spec)	-	Unif[logit(0.998), logit(0.9999)]
$r1_{\text{const}}$	day^{-1}	$\text{Unif}[0, 10^{-3}]$
log(c_2)	-	Gamma$(1, 1)$ in [0,log(50)]
Δr_1	year^{-1}	$\text{Unif}[0, 2.5]$
Δr_2	year^{-1}	$\text{Unif}[0, 2.5]$
x_0	-	$\text{Gamma}(10, 0.06)$ in $[0, 19]$
α_{pd}	-	$\text{Unif}[0.1, 5]$
κ_{as}	-	$\text{Gamma}(23.5, 3)$
κ_{pd}	-	$\text{Gamma}(23.5, 3)$

Table 3: Priors for fitted parameters. Non-uniform priors were additionally truncated with values given in brackets. Gamma priors are written with arguments of shape and scale.

Table 2: Model parameterisation (fixed parameters). Notation, a brief description, and the used values of fixed parameters.

Notation	Description	Value	
α	Rate at which tsetse become non-teneral (i.e. cannot get infectious)	73 year^{-1}	assumed
A/H_1	Density of non-human hosts relative to humans in area L	1.35	[3]
A/H_2	Density of non-human hosts relative to humans in area H	1.5	[3]
b	Proportion of infective bites leading to infection in humans	0.433	[3]
c_h	Proportion of bites on an infective human that lead to a mature infection in flies	0.065	[9]
c_{ai}	Proportion of bites on an infective non-human hosts of type i that lead to a mature infection in flies	0	
δ	Rate at which treated humans return to the susceptible class	2.19 year^{-1}	[10]
η	Rate at which hosts move from the incubating stage	31.025 year^{-1}	[9]
f	Inverse of duration of feeding cycle; or biting rate	121.545 year^{-1}	[11]
γ	Rate of progression to stage 2 in humans	0.6939 year^{-1}	[12]
μ	Death rate of humans due to natural causes	0.01666 year^{-1}	[13]
μ_γ	Disease-induced death rate or rate of leaving the recovered state for humans	1.4484 Year^{-1}	[12]
μ_t	Death rate of humans due to treatment	0 year^{-1}	assumed
μ_v	Death rate of tsetse	10.95 year^{-1}	[9]
ν	Inverse of the extrinsic incubation period	12.41 year^{-1}	[14]
σ	Biting preference for humans	0.326	[3]
σ_{ai}	Biting preference for non-human host in the setting i	0.8/0.396	assumed
ξ	Proportion of time spent i the high risk region by commuters	0.698	[3]
sensitivity	Diagnostics sensitivity (active screening)	0.91	[15]

Table 4: Model parameterisation (posteriors of fitted parameters). Notation, a brief description, and representative percentiles of the posterior distributions for fitted parameters. Here logarithm always refers to the natural logarithm.

Notation	Description	Posterior (median [95% CI])
κ	Ratio of humans in the high- to low-exposure environment	8.25 [3.82,11.6]$\times 10^{-2}$
log(VHL)	Log ratio of vectors to humans in low-exposure environment (VHL)	1.053 [0.974, 1.445]
log(c_1)	Log ratio of the ratio of vectors to humans in the high exposure environment to the ratio of vectors to humans in the low exposure environment	6.585 [0.547, 17.02]$\times 10^{-2}$
spec*	Diagnostic specificity (active screening)	0.9991 [0.9990, 0.9992]
$r1_{\text{const}}$	Daily passive detection rate for stage 1 (pre-2000)	3.62 [1.92,5.78]$\times 10^{-4}$
log(c_2)	Log ratio of passive detection for stage 2 to stage 1 (pre-2000)	0.559 [0.036, 1.773]
Δr_1	Amount passive detection in stage 1 improves	9.978 [4.402, 17.16]$\times 10^{-2}$
Δr_2	Amount passive detection in stage 2 improves (in addition to improvement of stage 1)	1.444 [0.646, 2.314]
x_0	Turning point (years since 1999) for logistic improvement in passive detection	13.24 [6.44, 18.76]
α_{pd}	Steepness in logistic improvement of passive detection	0.578 [0.182, 0.962]
κ_{as}	Overdispersion parameter (active screening)	34.04 [19.56, 56.49]
κ_{pd}	Overdispersion parameter (passive detection)	61.45 [39.13, 91.33]

* The specificity parameter, spec, was sampled in the logit scale, however posterior estimates are shown here in the model scale for clarity.

2.2 MCMC outputs

The MCMC outputs shown in Figures 2 correspond to 10,000 post burn-in samples.

2.2.1 Posterior densities

.

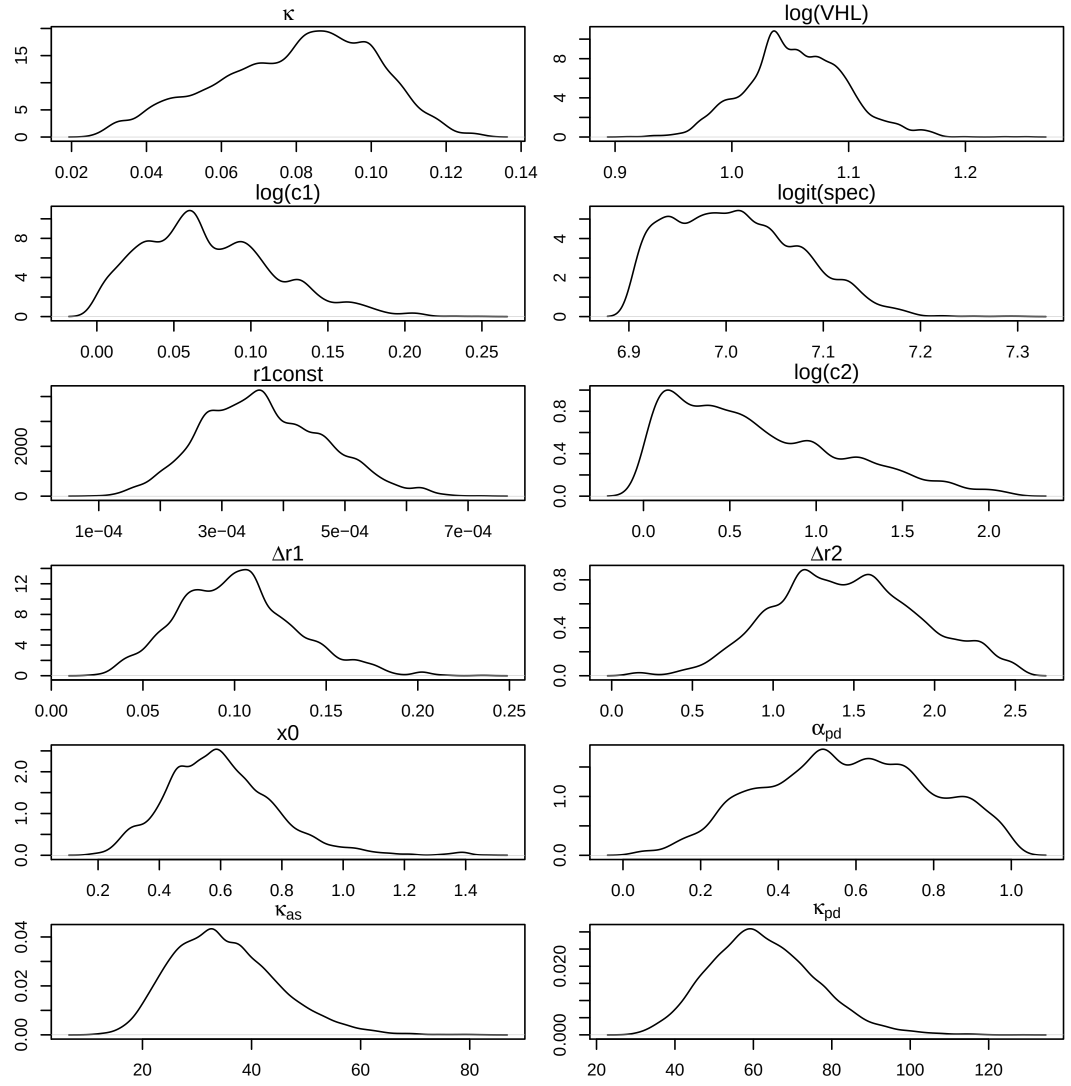

Figure 2: Posterior density of fitted parameters for Mushie territory.

2.2.2 Model fit to reported case data

Figure 3 shows the model fit to Mushie territory. The deterministic model simulations used 10,000 MCMC posterior samples.

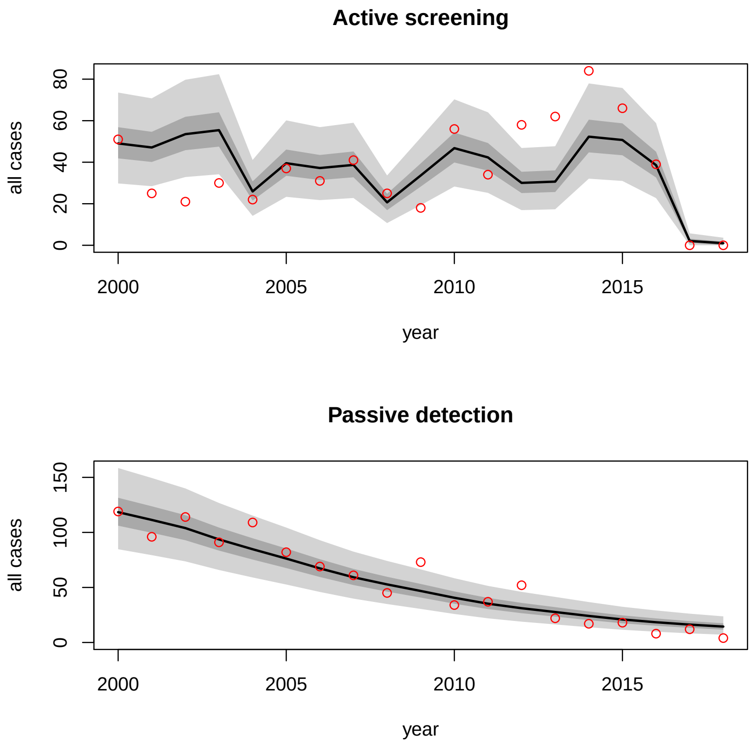

Figure 3: Model fit to reported case data for Mushie territory. Shaded regions indicate (2.5,97.5) and (25,75) percentiles.

References

[1] María Soledad Castaño, Martial L Ndeffo-Mbah, et al. Assessing the impact of aggregating disease stage data in model predictions of human African trypanosomiasis transmission and control activities in Bandundu province (DRC). *PLoS Neglected Tropical Diseases*, 14(1):e0007976, 2020.

[2] Maryam Aliee, Soledad Castaño, et al. Predicting the impact of COVID-19 interruptions on transmission of gambiense human African trypanosomiasis in two health zones of the Democratic Republic of Congo. *Transactions of The Royal Society of Tropical Medicine and Hygiene*, 115(3):245–252, 2021.

[3] Chris M Stone and Nakul Chitnis. Implications of heterogeneous biting exposure and animal hosts on *Trypanosomiasis brucei gambiense* transmission and control. *PLoS Computational Biology*, 11(10):e1004514, 2015.

[4] Kat S Rock, Martial L Ndeffo-Mbah, et al. Assessing strategies against Gambiense sleeping sickness through mathematical modeling. *Clinical Infectious Diseases*, 66(suppl_4):S286–S292, 2018.

[5] Daniel T Gillespie. Exact stochastic simulation of coupled chemical reactions. *The Journal of Physical Chemistry*, 81(25):2340–2361, 1977.

[6] Marc Artzrouni and Jean-Paul Gouteux. A compartmental model of sleeping sickness in central Africa. *Journal of Biological Systems*, 4(04):459–477, 1996.

[7] OCHA Office for the Coordination of Humanitarian Affairs. *Journées Nationales de Vaccination (JNV) Activités de vaccination supplémentaire , RDC*, Accessed May 2016.

[8] Simon E. F. Spencer. Accelerating adaptation in the adaptive Metropolis-Hastings random walk algorithm. *Australian and New Zealand Journal of Statistics*, In press.

[9] DJ Rogers. A general model for the African trypanosomiases. *Parasitology*, 97(1):193–212, 1988.

[10] Alain Mpanya, David Hendrickx, et al. Should I get screened for sleeping sickness? A qualitative study in Kasai province, Democratic Republic of Congo. *PLoS Neglected Tropical Diseases*, 6(1):e1467, 2012.

[11] World Health Organization and WHO Expert Committee on the Control and Surveillance of Human African Trypanosomiasis. Control and surveillance of human African trypanosomiasis. Technical Report 984, World Health Organization, November 2013.

[12] Francesco Checchi, Sebastian Funk, et al. Updated estimate of the duration of the meningo-encephalitic stage in gambiense human African trypanosomiasis. *BMC Research Notes*, 8(1):292, 2015.

[13] The World Bank. *Data: Democratic Republic of Congo*, Accessed 2015.

[14] Sophie Ravel, Pascal Grébaut, et al. Monitoring the developmental status of *Trypanosoma brucei gambiense* in the tsetse fly by means of PCR analysis of anal and saliva drops. *Acta Tropica*, 88(2):161–165, 2003.

[15] Francesco Checchi, François Chappuis, et al. Accuracy of five algorithms to diagnose gambiense human African trypanosomiasis. *PLoS Neglected Tropical Diseases*, 5(7):e1233, 2011.
